# A Novel Approach to Pattern Dermal Papilla Spheroids in Dermal–Epidermal Composites Using Non-Adherent Microwell Arrays

**DOI:** 10.3390/bioengineering12121281

**Published:** 2025-11-21

**Authors:** E. Cate Wisdom, Donald C. Aduba, Owen Lewis, Sandhya Xavier, Ernest O. N. Phillips, Kristin H. Gilchrist, Ira M. Herman, Vincent B. Ho, Thomas N. Darling, George J. Klarmann

**Affiliations:** 1 USU Center for Biotechnology (4DBio^3^), Department of Radiology and Bioengineering, Uniformed Services University of the Health Sciences, Bethesda, MD 20814, USA; emily.cate.wisdom@gmail.com (E.C.W.); olewis@genevausa.org (O.L.); khgilchrist@gmail.com (K.H.G.); vincent.ho@usuhs.edu (V.B.H.); 2 The Geneva Foundation, Tacoma, WA 98402, USA; 3 Department of Dermatology, Uniformed Services University, Bethesda, MD 20814, USA; donald.aduba.ctr@usuhs.edu (D.C.A.J.); sandhya.xavier.ctr@usuhs.edu (S.X.); ernest.phillips.ctr@usuhs.edu (E.O.N.P.); thomas.darling@usuhs.edu (T.N.D.); 4 The Henry Jackson Foundation, Bethesda, MD 20817, USA; 5 Department of Developmental, Molecular, and Chemical Biology, Tufts University School of Medicine, Boston, MA 02111, USA; ira.herman@tufts.edu; 6 Center for Innovations in Wound Healing Research, Tufts University School of Medicine, Boston, MA 02111, USA; 7 Tissue Health Plus, Inc., Fort Worth, TX 76102, USA

**Keywords:** 3D printing, biofabrication, hair follicle neogenesis, dermal papilla, skin composite, graft survival, regenerative medicine, wound healing

## Abstract

Bioengineered dermal–epidermal composites (DECs) have demonstrated promise initiating skin regeneration and hair follicle neogenesis after injury. DECs in our work comprise a collagen matrix embedded with human dermal papilla cells (HDPCs) overlaid with human keratinocytes. HDPCs, as three-dimensional spheroids, enhance hair follicle formation, working in tandem with keratinocytes. Herein, 3D printed stamped PDMS microwell arrays were used as a strategy for spatially patterning dermal papilla spheroids in the dermal components of the DEC. DECs were transferred to cell culture media for 5 days followed by air–liquid interface culture for 2 days. Spheroid diameter, cell viability, and qPCR gene expression analyses were conducted. DECs were surgically grafted on immunocompromised mice, and healing was followed for 10 weeks. HDPCs cultured in the microwell arrays formed patterned viable spheroids and successfully transferred to the collagen dermal matrix. RNA analysis using qPCR showed upregulation of key HDPC markers (*VCAN* and *BMP6*) in DC microwell patterned HDPC spheroids compared to monolayers. This work represents a novel 3D printing strategy optimizing designing patterned HDPC spheroids in the extracellular matrix to regenerate functional human skin instead of scar tissue after injury.

## 1. Introduction

Wound management strategies to treat skin loss from burns, trauma, and disease remain a public health concern in the United States as medical treatment costs continue to increase. A published study in 2023 reported wound care patients on Medicare increased from 8.2 to 10.5 million from 2014 to 2019, respectively [1]. Globally, the wound care market is projected to reach over USD 18.5 billion by the year 2027 [2]. Non-healing wounds have greater incidence than all cancers combined according to a 2022 review published by Weigelt et al. [3]. This dilemma puts significant strain on clinicians to effectively diagnose, treat, and manage wounds in the current healthcare landscape [3]. Given the accelerating demands on hospitals, viable skin grafts that efficiently heal full-thickness wounds are critical to reduce the burden on clinicians.

Traditionally, skin grafting involves using autografts, where skin is harvested from the patient’s own body, or allografts, where skin is obtained from deceased donors. While effective in healing wound injuries, these methods present challenges such as availability, cost, donor site discomfort, risk of rejection, and scarring [4,5,6,7,8]. Split-thickness skin grafts are composed of an epidermal layer and part dermal layer from the donor tissue. Split-thickness skin grafts are considered the current “gold standard” due to its ability to be meshed, covering large wound areas and draining fluid out of the wound bed to minimize infection and encourage complete wound epithelialization [6,9,10]. While split-thickness skin grafts offer those properties, hair follicle neogenesis is limited, leading to scarring and incomplete healing [11].

Tissue-engineered skin grafts offer a promising alternative. These grafts can be customized with growth factors, key reparative and regenerative peptides, cells, and materials to potentially reduce donor site morbidity, lower the risk of rejection, and improve integration and functionality [12,13,14,15,16,17]. Tissue-engineered skin represents a versatile solution not wholly reliant on donor skin compared to traditional grafting methods. Skin composites engineered to contain neonatal fibroblasts and keratinocytes as respective dermal and epidermal layers in a collagen matrix have been approved by the U.S. Food and Drug Administration (FDA) for treatment of diabetic and venous foot ulcers [8]. While many advances have been made developing tissue-engineered skin grafts, little progress has been made towards inducing hair follicle formation during wound repair.

A critical component of skin function during wound repair is enabled by human dermal papilla cells (HDPCs), a subtype of fibroblasts that reside at the base of the hair follicle. Dermal papilla cells are essential for hair follicle formation by enhancing signaling between the epithelium and mesenchymal skin components during the hair cycling process [18,19]. HDPCs’ impact on hair follicle growth contributes to skin integrity and minimizing scar formation, which is critical for replicating natural skin properties [20]. Dermal–epidermal composites (DECs) with optimally dissociated DPCs cultured in 2D have been successfully grafted into a rodent model, with over 80% of those grafted composites demonstrating hair follicle neogenesis [21]. DECs have also been incorporated with bioactive peptides with reparative and regenerative properties to induce good graft survival, increase epidermal thickness, and increase the hair follicle number in rodent models [16].

Monolayer HDPC culture in vitro requires a large population of cells passaged multiple times, leading to loss in hair follicle inductivity [22]. Therefore, HDPCs have been investigated to develop 3D cellular spheroids using the hanging drop method to mimic the 3D microenvironment, demonstrating greater hair follicle inductivity compared to 2D culture environments [23,24]. While this approach has been used to generate 3D spheroids in vitro [25] and in vivo [26] as grafted skin composites, challenges exist to rapidly integrate HDPC spheroids into tissue-engineered skin composites for grafting due to the length of time (≥2 weeks) it takes to prepare the grafts in culture prior to grafting onto the mouse.

**Figure 1:**
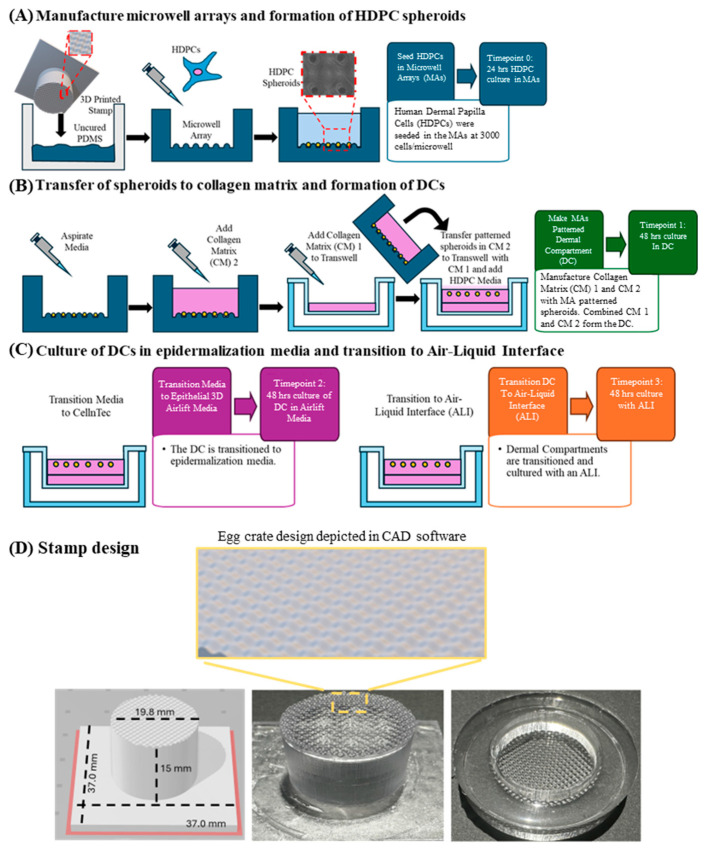
Schematic overview of procedure and experimental design. (**A**) Depiction of microwell array (MA) fabrication from stereolithography 3D printed stamps. The MAs were steam sterilized, and singularized human dermal papilla cells (HDPCs) were seeded at a density of 3000 cells/microwell. (**B**) Depiction of HDPC spheroid transfer out of the MAs in a collagen matrix (CM) to a Transwell cell culture system. (**C**) Depiction of in vitro DC culture transitions in preparation for grafting onto mice. (**D**) Depiction and dimensions of 3D printed egg crate stamp and the resulting PDMS microwell array (from left to right).

In our work, we employed 3D printing stereolithography to create microfabricated stamps generating non-adherent microwell arrays that support patterned spheroids in collagen DECs (Figure 1). This work combines the advantages of culturing HDPC dermal papilla spheroids with 3D printing to enhance tissue-engineered DECs. Compared to the state of the art in employing spheroids into skin composites, DECs fabricated using non-adherent microarrays in this work enable HDPC spheroid formation from singularized cells and rapid production times (~1 week) before grafting. DECs fabricated using our technique exhibited consistent patterned HDPC spheroid formation and distribution and increased gene expression of the HDPC-niche markers *FGF*, *VCAN*, and *BMP6*, which are needed for hair follicle neogenesis. DECs with spatially patterned spheroids using microwells were grafted into a rodent model, exhibiting graft acceptance up to 10 weeks in vivo. Based on these findings, using non-adherent microwell arrays to spatially pattern dermal papillae in skin composites offers a tangible alternative to more rapidly address demands to heal full-thickness wounds by fulfilling the skin’s aesthetic and functional needs.

## 2. Methods

### 2.1. HDPC Culture

Human dermal papilla cells (HDPCs) (PromoCell, Heidelberg, Germany) were cultured in complete Follicle Dermal Papilla Cell Growth Medium (HDP-M) (PromoCell, Heidelberg, Germany) supplemented with 1% Penicillin–Streptomycin (Gibco, Grand Island, NY, USA), 0.1% Gentamicin (Gibco), and 0.2% Amphotericin (Gibco). HDPCs were seeded in tissue culture flasks at a density of 5000 cells/cm^2^ and cultured to a confluency of 80% prior to passaging or seeding in microwell arrays.

### 2.2. Design and Fabrication of Microwell Arrays

A digital rendering of the 3D printed negative stamp was generated using computer-aided design (CAD) software (Autodesk Inventor Professional v2023.2, Build 158, San Francisco, CA, USA). The CAD rendering was exported as a standard triangle language (.stl) file and uploaded into slicing software (PreForm version 3.37.5, Formlabs, Sommerville, MA, USA) to be printed on a stereolithography 3D printer (Form3B, Formlabs) using a biocompatible clear resin (BioMed Clear, Formlabs). Following printing, the stamps were rinsed in 99% pure isopropanol for 20 min and allowed to air-dry in a light protected drawer. The dry parts were then photocured for 60 min at 60 °C in a light box (FormCure, Formlabs). The stamp generates 257 wells and is 19.8 mm in diameter post-curing. Each well is 0.5 mm deep (peak to valley). The design is a sine wave pattern in both the x and y direction, with wells spaced 1 mm apart center to center in the x and y directions (see Figure 1D for images and dimensions). The resulting DP spheroid density equates to 83 spheroids/cm^2^ and falls in the range of average measured density of 14–292 hair follicles/cm^2^ in human skin [27]. Microwell arrays were fabricated by inserting the 3D printed negative stamp into uncured Polydimethylsiloxane (PDMS) (Dow Sylgard 184, Ellsworth Adhesives, Germantown, WI, USA) or molten low-gelling-temperature agarose Type VII-A (Thermo Fisher, Waltham, MA, USA). The PDMS was cured in an 80 °C oven for three hours and then autoclave-sterilized prior to seeding cells. The molten agarose was pipetted into a 6-well plate in a tissue culture hood and allowed to solidify in the hood to maintain sterility.

### 2.3. HDPC Spheroid Formation with Hanging Drop Method and Non-Adherent Microarray Method

Rat Tail Collagen I at a concentration of 0.1 mg/mL (Corning, Glendale, AZ, USA) was used in a 10 µL suspension of 3000 HDPCs in HDP-M and pipetted on the underside of the lid of a plastic Petri dish, typically with 64 individual droplets. Dishes contained 2 mL of sterile water, and lids were carefully placed on the dish. These were incubated in a humidified 37 °C cell incubator for 24 h to generate spheroids. To make spheroids in the microwells, HDPCs were passaged using 0.04% Trypsin/0.03% EDTA (Detach kit, PromoCell). Detached HDPCs were centrifuged and resuspended at a concentration of 514,000 cells/mL. The sterile microwell arrays were placed in a 35 mm Petri dish or 6-well plate. Then, 1.5 mL of the HDPC suspension was pipetted onto the microwell array (Figure 1A). Two non-adherent substrates were first compared to see if singularized HDPCs formed spheroids within microwells made of either agarose or PDMS. The Petri dish containing the microwell array was transferred to a cell culture incubator at 37 °C, 5% CO_2_, for 24 h.

### 2.4. Biofabrication of DCs and DECs Using Spheroids from Non-Adherent Microarrays

This process is illustrated in Figure 1. Dermal compartments were biofabricated by using a Rat Tail Collagen I (Corning)-based master mix (Figure 1B). The master mix is composed of the following: 10× DMEM (81 µL/mL), Glutamax (8.1 µL/mL), Fetal Bovine serum (90 µL/mL), 71.2 mg/mL Sodium Bicarbonate (55.5 µL/mL), and Gentamicin (1 µL/mL) (all from Thermo Fisher, Frederick, MD, USA). The final concentration of Rat Tail Collagen I was 1.2 mg/mL. Prior to use, the master mix solution was kept in a 4 °C refrigerator. Each aliquot of master mix was pH tested using a 10 µL sample and a pH strip to ensure a pH range of 7.0–7.4 was achieved. A 1 mL layer of the master mix was applied to a collagen I coated Transwell cell culture Polyester insert surface with a 3.0 µm pore size (BioCoat^®^, Corning). This “CM1” acellular layer was placed in the 37 °C incubator for 30 min to allow gelation to occur. Microwell arrays seeded with “singularized” HDPCs 24 h prior (Figure 1A) were removed from the incubator and spheroid formation was visually confirmed using light microscopy (Nikon Eclipse Ts2 (Nikon Instruments, Inc., Melville, NY, USA). Under sterile conditions, the medium from the microwell array was carefully aspirated in 200 µL increments. Once the medium was completely removed from the microwell array, 1.5 mL of master mix was gently added to the array and allowed to gel in the 37 °C incubator for 30 min to create the “CM2” layer. After both the CM1 layer and the microwell gelled, 500 µL of master mix was added to the top of CM1 in the Transwell to facilitate layer adherence; then, the microwell was removed from the Petri dish, and while hovering over the recipient Transwell, the microwell sides were pinched gently to encourage the “CM2” cellular layer to transfer into the Transwell. Once the transfer was complete, an additional 1 mL of master mix was pipetted over the CM2 layer to ensure cohesion between the layers (Figure 1B). The microwell was placed in the 37 °C incubator for 30 min to allow gelation. Following gelation, 2 mL of Promocell HDP-M is added to the basal side of the Transwell insert followed by the addition of 1 mL of medium to the apical side of the insert. After 48 h, HDP-M is changed to specialized epithelial media (CnT-Prime Epithelial 3D Airlift Medium, CellnTec Advanced Cell Systems, Bern, Switzerland) (PA) in preparation of the DC to transition to the air–liquid interface step before harvesting DCs for RNA analysis (Figure 1C). Note that human keratinocytes (HKs) were only added to DECs used for skin grafting, and DCs for RNA analysis did not use HK cells.

**Figure 2:**
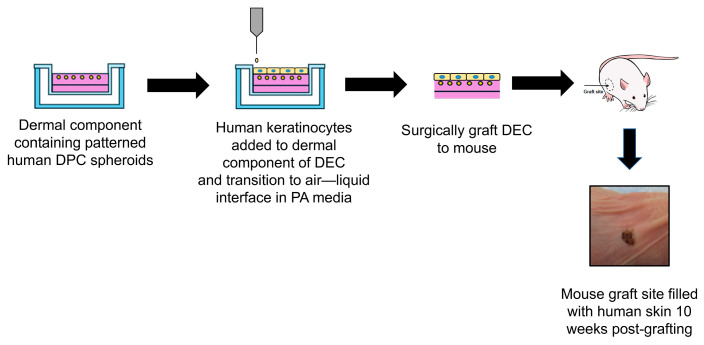
Schematic of adding epidermal layer of keratinocytes to dermal component of DECs before transition to air–liquid interface in PA cell culture media. DECs are subsequently grafted into a wound area laterally located on the ventral side of immunocompromised mice (image shown).

In DECs prepared for grafting, HKs were seeded on the top portion of the array prior to adding PA media. Following propagation, HK cultures were lifted, collected, and pelleted. The supernatant was removed and replaced with PA media. HKs were resuspended in PA media at a concentration of 1,000,000 cells/mL. Before seeding HKs, HDP-M was aspirated from the basal and apical sides of the DC Transwell insert. The basal surface was replenished first with 2 mL of PA media. The apical portion received 1.5 mL of the HK suspension. The DECs were placed into the 37 °C incubator. After 48 h in culture following HK seeding, the DEC was transitioned to the air–liquid interface by firstly removing the media from both the apical and basal sides of the Transwell inserts. The apical side was left exposed with no media, while the basal side was replenished with 2 mL of PA media. The DECs were placed into the 37 °C incubator, with media changes occurring every 48 h until harvesting the DECs for surgical grafting into an immunocompromised mouse model (Figure 2).

### 2.5. Imaging and Characterization of HDPC Spheroids in Microwell Arrays and Dermal Compartments

DCs, prior to confocal analysis, were imaged using light microscopy. DCs were removed from Transwell inserts and placed in a Petri dish with approximately 500 µL of PA media to prevent desiccation. The images were collected and analyzed using Nikon NIS-elements software v5.21.03. HDPC spheroid cell viability within biofabricated DCs was evaluated using a cell viability/cytotoxicity assay (Biotium, Freemont, CA, USA). A staining solution of DPBS, 2 µM Calcein AM, and 4 µM ethidium homodimer-III was used. DCs were placed in a Petri dish and submerged into the staining solution. The Petri dish was carefully wrapped in aluminum foil and placed in an incubator at 37 °C for 30 min. The staining solution was aspirated followed by a DPBS wash, and 500 µL of PA media was added to maintain hydration throughout the imaging process. The sample was then placed on the Leica DMi8 inverted confocal microscope (Leica Microsystems, Boston, MA, USA). Images were collected of the entirety of the DC to visualize spheroid patterns and determine the location of any diffuse cell populations. Z-Stacks were acquired of individual spheroids and spheroid clusters.

### 2.6. RNA Isolation

RNA from HDP cells in monolayer culture, as spheroids or from DECs lacking keratinocytes, was isolated according to manufacturer’s instructions using the RNeasy Plus Micro kit and the use of genomic DNA (gDNA) removal columns (Qiagen, Germantown, MD, USA). To isolate RNA from HDPC spheroids embedded in the collagen dermal composite, one-half of the construct was placed into 1 mL of Trizol (Thermo Fisher) and incubated with gentle agitation for up to 20 min at room temperature until the DEC was solubilized. Chloroform (Sigma, St Louis, MO, USA) (0.2 mL) was added, and the mixture was vortexed. Following a 3 min room temperature incubation, the material was centrifuged at 12,500× *g* at 4 °C for 15 min. Following centrifugation, the upper aqueous phase was removed, and one volume of 70% ethanol was added. Purification was then achieved with an RNeasy Plus Micro kit, but gDNA removal columns were not utilized. In all cases, RNA was eluted with RNase-Free H_2_O and stored at −80 °C.

### 2.7. cDNA Reactions and qPCR Gene Expression

Using an input of 60 ng of RNA, cDNA was generated in 20 µL reactions with Superscript Vilo IV MasterMix cDNA kits (Thermo Fisher) according to manufacturer’s instructions. qPCR was performed with TaqMan Fast Advanced MasterMix (Thermo Fisher) in a 10 µL reaction volume with an input of 1 µL of cDNA in a MicroAmp EnduraPlate Optical 96-Well plate (Thermo Fisher) in a 0.2 mL block in the QuantStudio 3 machine (Thermo Fisher). Cycle parameters were 50 °C for 2 min, 95 °C for 20 s, then 40 cycles of 95 °C for 1 s, and 60 °C for 20 s with fast cycling and using ΔΔCt mode. ROX was used as passive reference dye to normalize fluorescent qPCR signals, and Ct values were normalized to those of GAPDH [28,29]. Three biological replicates and two technical replicates of each sample (n = 6) were used to calculate the relative quantitation (RQ) of spheroids to DP cell monolayers using QuantStudio v1.5.2 software. R software (v 4.3.2; R Foundation of Statistical Computing, Vienna, Austria) was used to calculate the Log2 Fold change in hanging drop and microwell spheroids compared to the DP cell monolayer. Gene expression during DC cell culture was measured across 1-, 3-, 5-, and 7-day timepoints in microwell patterned HDPC spheroids compared to the monolayer. All primers targeted human gene sequences and were validated TaqMan assays (Thermo Fisher) that amplify across exon junctions and should exclude gDNA amplification. The following genes and assay IDs were used: ALPL, Hs01029144_m1; ACTA2, Hs05005341_m1; VCAN, Hs00171642_m1; BMP6, Hs01099594_m1; FGF7, Hs00940253_m1; WNT5a, Hs00998537_m1; LEF1, Hs01547250_m1; AXIN2, Hs00610344 m1; GAPDH, Hs99999905_m1; and HIF1a, Hs00153153_m1.

### 2.8. Dermal–Epidermal Composite Grafting

DECs were grafted onto 7-week-old NIH(S)-nu/nu female mice (n = 4) (Charles River Laboratories, Wilmington, MA, USA). The bandaging protocol was modified from our previous work [21]. Mouse backs were swabbed with Betadine, then with hydrogen peroxide, before drying with cotton gauze. Next, an incision slightly larger than the size of the graft was made. Following the incision, the graft was inserted into the wound site and then secured using a Vaseline™ Petroleum gauze strip (COVIDIEN™, Medtronic, Minneapolis, MN, USA) followed by a flexible fabric Band-Aid. The graft was then secured using a Coban™ strip (3M, Saint Paul, MN, USA) and a sheer strip Band-Aid. Two weeks after the initial grafting, the entire bandage was removed, with the surgical area cleaned with hydrogen peroxide, dried, and then secured by a Vaseline™ Petroleum gauze strip (COVIDIEN™). A Telfa™ (COVIDIEN) strip was placed on top of the Vaseline gauze strip and secured by medical adhesive tape (Micropore, 3M), followed by a Coban™ strip (3M) and a sheer strip Band-Aid. The bandages were completely removed after 4 weeks. Gross imaging of the rodent graft acceptance was carried out at 4 w, 6 w, 8 w, and 10 w before the animals were euthanized. Tissue was harvested, formalin-fixed, or frozen for histological analysis. The Nikon Eclipse Ti microscope (Nikon Americas Inc., Melville, NY, USA) with a Nikon DS-Ri2 color CMOS camera scanned H&E-stained graft tissue sections from microwell array DECs using a 4× objective lens. All animal experiments were performed in accordance with our Institutional Animal Care and Use Committee (IACUC) guidelines.

### 2.9. Statistical Analysis

The statistical analysis was performed using a two-tailed unpaired *t*-test and one-way ANOVA with Tukey’s post hoc test using R software. Data are presented as the mean ± standard deviation reported in the spheroid diameter and distance figures. Data are presented as the mean ± standard error in qPCR gene expression figures. *p*-values < 0.05 were denoted as statistically significant.

## 3. Results

### 3.1. HDPC Spheroid Formation with Hanging Drop Method

**Figure 3:**
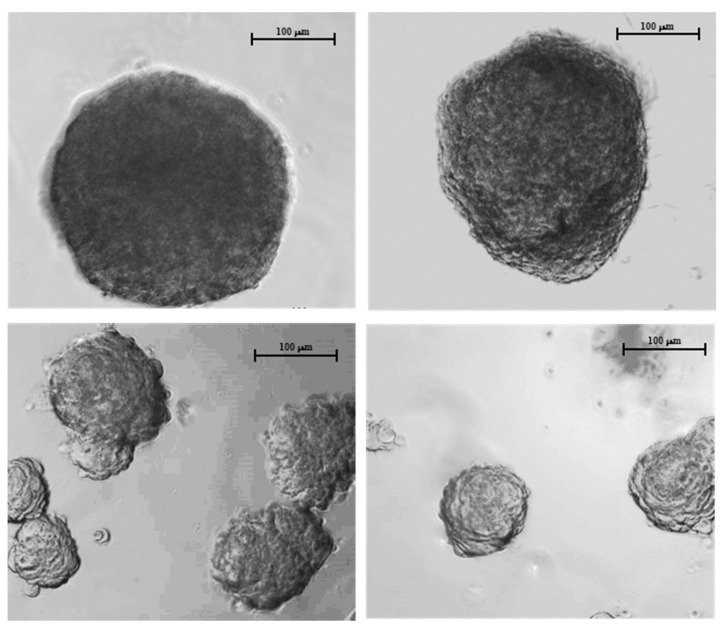
Representative human dermal papilla cell spheroids formed using the hanging drop method. Top row: Here, 0.1 mg/mL Corning Rat Tail Collagen I was added to a 10 µL suspension of 3000 HDPCs and cultured for 24 h. Bottom row: No collagen was added to the cells. Scale bar = 100 µm.

HDPCs were mixed with collagen and formed spheroids in a hanging drop (Figure 3, top row). Rat Tail Collagen was pre-screened for the ability to facilitate spheroid formations. The spheroids composed of 3000 cells exhibited a diameter of 348 ± 15.6 µm. Typically, spheroids form in nearly every hanging drop. In contrast, when Rat Tail Collagen was omitted, no spheroids were formed. Instead, cell aggregates of predominantly two sizes were detected (Figure 3, bottom row). One group was 106 ± 1.24 µm, while the other was 149 ± 16.8 µm. These aggregates lacked the smooth surface appearance of true spheroids.

### 3.2. HDPC Spheroids Pipetted into Microwell Array

**Figure 4:**
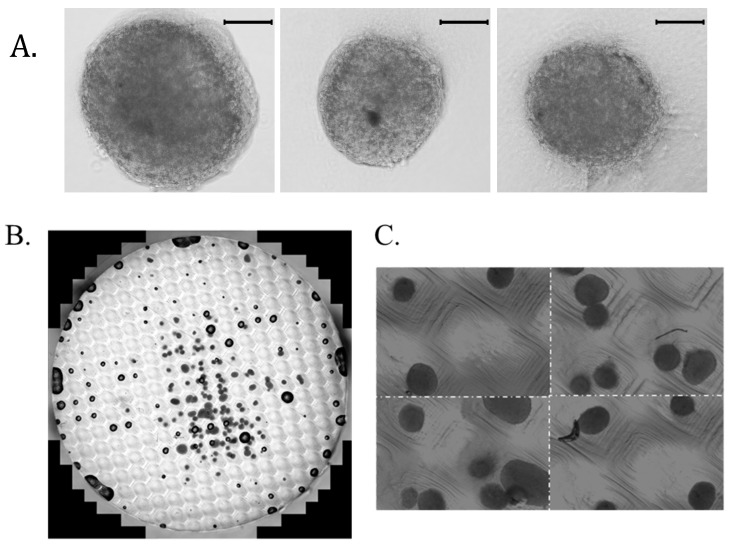
Premade hanging drop spheroids: scale bar 100 microns (**A**), trans PMT image of the entire DC that was made via pipetting premade hanging drop spheroids; array diameter is 19.9 mm (**B**), close-up view (zoomed in) of the spheroids in the microwell array illustrating the lack of uniformity. Spheroids are approximately 300–350 microns in diameter (**C**).

To pattern the HDPC spheroids within the collagen dermal composites, hanging drop spheroids were made and collected. They were mixed directly via pipetting with the collagen liquid (Figure 4A) and permitted to gel, or they were collected and pipetted into a non-adherent, PDMS egg crate-design microwell array, allowed to settle for an hour, and then overlaid with collagen (Figure 4B). While there was a distribution of spheroids throughout the construct, there is no discernable patterning. Even when allowed to settle in the premade troughs of the microwells, the spheroids were not evenly distributed within the microwells (Figure 4C). The microwells that contained single spheroids do exhibit the proper spacing of the array pattern. However, making dermal composites in this manner would make it difficult to generate repeatable spheroid distribution pattern results, and further optimization and refinement of the process was needed. Thus, a new method was employed where singularized cells were seeded into arrays made of agarose or PDMS to form spheroids de novo.

### 3.3. Patterning HDPC Spheroids from Singularized Cells in Non-Adherent Microarrays

**Figure 5:**
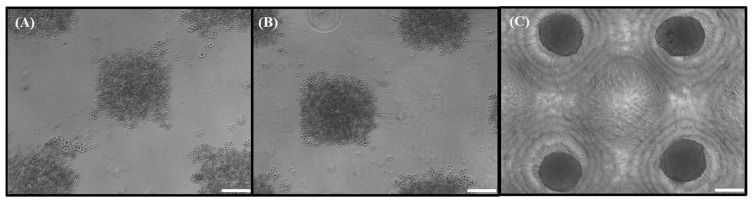
Singularized HDPC response to culture in egg crate-designed microwell arrays fabricated from different non-adherent substrate materials. Stereolithography 3D printed stamps with an egg crate microwell pattern were used to mold 2% agarose (**A**), 5% agarose (**B**), and PDMS (**C**) substrate materials. HDPCs at 3000 cells per microwell were added to the arrays, cultured for 24 h, and imaged using light microscopy. The softer 2% and 5% agarose hydrogel substrate materials did not result in spheroid formation but did effectively pattern the singularized HDPCs. The stiffer silicone elastomer PDMS microwell arrays did result in HDPC spheroid formation and patterning when seeded with singularized HDPCs. Scale bars = 200 µm.

Two non-adherent substrates were first compared to determine if singularized HDPCs formed spheroids within microwells made of either agarose or PDMS (Figure 5). In all cases, the cells settled into the wells and coalesced into the designed pattern. In the agarose wells (Figure 5A,B), the cells were looser and made larger-diameter aggregates regardless of agarose concentration. HDPCs in PDMS wells (Figure 5C) patterned as desired and made smooth-looking spheroids that are similar in size to those formed in the traditional hanging drops.

### 3.4. Imaging and Characterization of HDPC Spheroids in Microwell Arrays and Dermal Compartments

**Figure 6:**
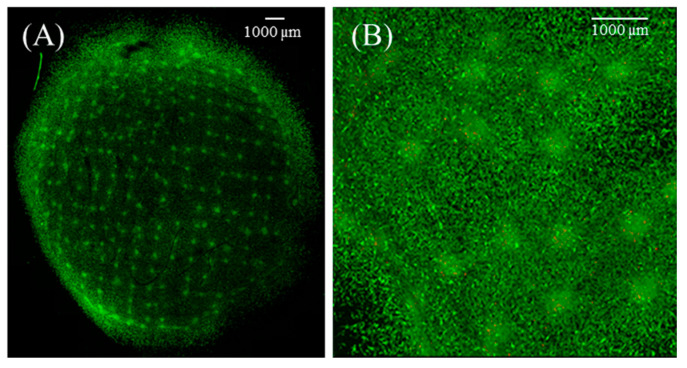
Patterned spheroids in the dermal composite. Confocal microscopy image of Calcein AM live-stained HDPC spheroids formed and patterned by the PDMS microwell arrays. (**A**) Images are of the whole dermal compartments after transition to the air–liquid interface. Spheroids appear patterned, and HDPCs are migrating throughout the collagen matrix. Confocal microscopy reveals significant HDPC viability, as indicated by the green staining. (**B**) Increased magnification better shows that HDPCs are spreading throughout the collagen matrix. Scale bars on both images are 1000 µm.

Briefly, singularized HDPCs were seeded directly onto the arrays in HDP cell media. The seeding density corresponds to 3000 cells/well to duplicate the hanging drop cell density. Unlike the hanging drops, Rat Tail Collagen type I was omitted from the media, as it was not necessary for de novo spheroid formation. After visual confirmation of the newly generated spheroids, HDPC medium was removed; then, collagen layer CM2 was deposited over the microwells. The collagen matrix containing HDPC spheroids was transferred to a Transwell insert with a layer of collagen already in place and transitioned to the air–liquid interface for culture. Calcein AM staining shows that the pattern is maintained across the entire Transwell, with viable cells on the surface of the spheroid (Figure 6). There was also evidence of HDPC outgrowth from the spheroids into the surrounding collagen matrix.

**Figure 7:**
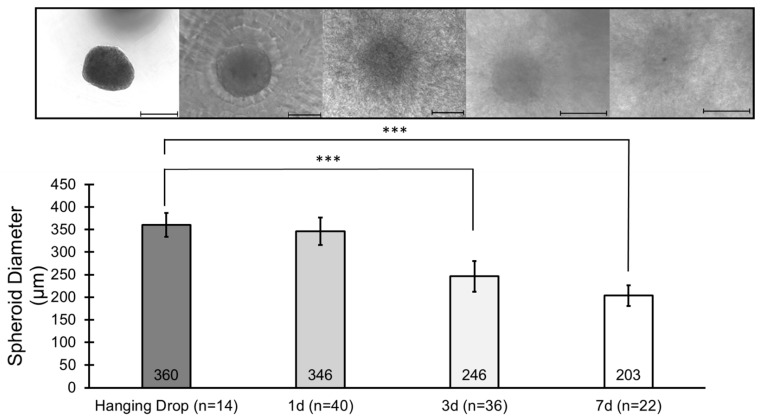
HDPC spheroid diameter decreases over time. Quantification of the initial and cultured diameters of spheroids formed in PDMS microwell arrays compared to spheroids formed with the traditional hanging drop method. The diameter of spheroids formed in the microwell arrays after 1 day is not statistically different from the diameter of spheroids formed by the hanging drop method. The spheroid diameter changes as the culture time increases. This can be attributed to cells migrating away from the spheroids into the surrounding collagen matrix. Scale bars = 200 µm. Significance comparing hanging drop spheroid diameter to microwell spheroid diameter at various timepoints is denoted at statistical levels: *** *p* < 0.001.

Because of the observed HDPC outgrowth, phase contrast microscopy was used to measure the diameter of the HDPC spheroids within the DC over time (Figure 7). As noted previously, spheroids initially measured 346 ± 31 µm at 1 day, which was equivalent in diameter to those formed in hanging drops. At 3 days, the diameter decreased to 246 ± 34 µm, and at 7 days, the spheroid diameter was 203 ± 23 µm. Along with the change in size, the appearance of the spheroids also changed, and they became more difficult to see due to the outgrowth of cells into the surrounding collagen matrix. Thus, the outgrowth of cells from the HDPC spheroids resulted in the gradual reduction in spheroid diameter during the culture period.

**Figure 8:**
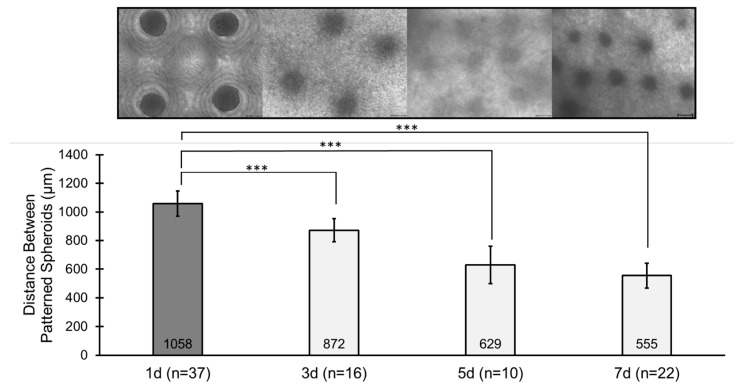
Distance between HDPC spheroids over time. Quantification of the distance of patterned HDPC spheroids formed in PDMS microwell arrays (1 d), transferred to the collagen matrix dermal compartment (3 d), cultured in epidermalization media (5 d), and transitioned to the air–liquid interface. The average distance of patterned spheroids decreases as the collagen matrix contracts and is reorganized by HDPCs. Scale bars = 200 µm. Note that the 1 d image on the top left is the same image shown in Figure 5C. Significance comparing microwell spheroid distance at 1 day to later timepoints is denoted at statistical levels: *** *p* < 0.001.

Similarly, the spacing between spheroids in the pattern was measured at each timepoint (Figure 8). The initial spacing at 1 d is 1058 ± 89 µm, which is the center-to-center spacing between adjacent wells in the PDMS micro-mold array. As the time in culture increased, the spacing between spheroids decreased nearly by half by 7 d. This aligns with the DEC contracting due to the cellular activity of HDPCs interacting with the collagen matrix. In this case, the DECs contracted to approximately half of their initial size. The spheroid pattern is still visible at 7 d.

### 3.5. qPCR Gene Expression Across DC Cell Culture Platforms

**Figure 9:**
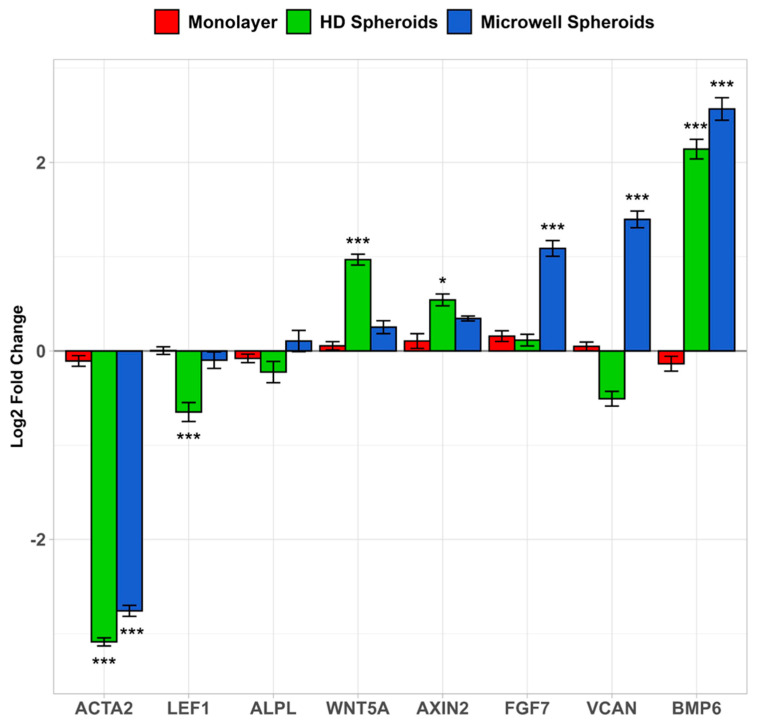
Log2 fold gene expression comparison of important HDPC niche and hair follicle markers in HDPC monolayer, hanging drop (HD), and microwell spheroids after 24 h culture. *ACTA2* gene served as a negative control. Significance comparing cell platforms to monolayers is denoted at statistical levels: * *p* < 0.05; *** *p* < 0.001.

Gene expression was used to compare dermal components of HDPC spheroids produced from the PDMS microwell molds to the traditional hanging drop method (Figure 9). A selection of eight genes linked to trichogenicity or whose expressions are known to change in 3D culture were examined. Since smooth muscle actin (*ACTA2*) expression decreases in 3D hanging drop culture compared to monolayer culture [22], it was used as a control to validate that our system works as predicted. After 24 h of spheroid formation, smooth muscle actin was downregulated up to 8.5-fold in hanging drop HDPCs. *BMP6* was upregulated 4.4-fold in HDPCs grown in hanging drops and 5.9-fold in microwell HDPC spheroids. *FGF7* and *VCAN* transcription increased over 2-fold in the microwell spheroids but not in HDPC hanging drop spheroids. *FGF7* RNA expression in the 3D microwell HDPC spheroids was higher than that of monolayers and hanging drop spheroids. *LEF1* in hanging drop spheroids was slightly downregulated (1.6-fold) compared to the HDPC monolayer. There was little to no change in expression of the other genes examined. Thus, with this eight-gene survey, the dermal papilla spheroids formed in the microwell molds appear to have gene expression levels more similar to the hanging droplet spheroids than the monolayer HDPCs.

### 3.6. qPCR Gene Expression Across DC Cell Culture Time Course

**Figure 10:**
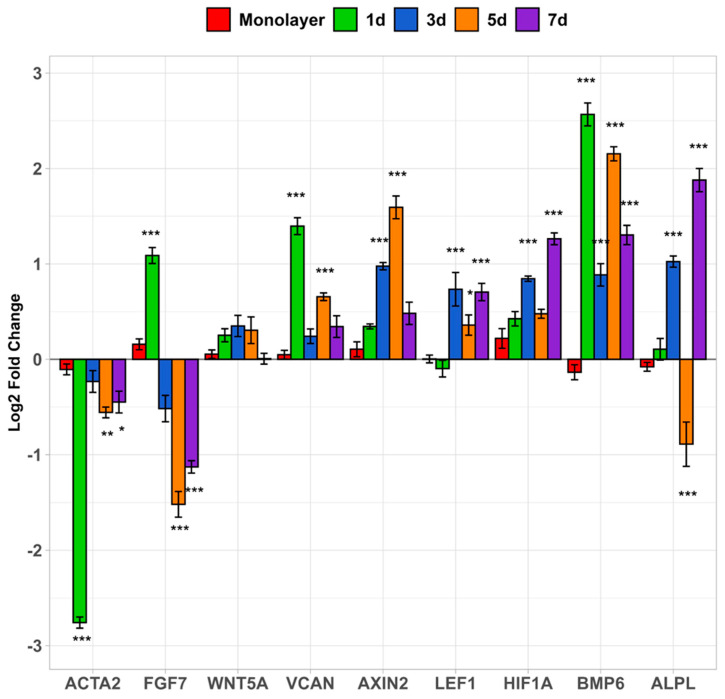
Log2 fold gene expression comparison of important HDPC niche and hair follicle markers in microwell HDPC spheroids in DC culture from 1 to 7 days. *ACTA2* gene served as a negative control. Significance comparing cell platforms to monolayers is denoted at statistical levels: * *p* < 0.05; ** *p* < 0.01; *** *p* < 0.001.

During the time course from patterned dermal compartments initially in growth medium to the 7-day timepoint culturing the DC in the air–liquid interface, gene expression was monitored to determine how the different growth medium conditions affect genes implicated in DP cell trichogenicity (Figure 10). There was a gradual increase in alkaline phosphatase (ALPL) gene expression as the culture time increased. However, at 5 days, when the medium was changed to PA media, *ALPL* significantly dropped compared to the monolayer and day 1 timepoints and then recovered again at 7 days. Compared to the expression within the microwell spheroids at 1 day, *BMP6* gene expression dropped in 3D cultures but still remained higher than the monolayer cultures after 7 days. *VCAN* expression fluctuated between 3 days and 7 days to return to levels similar to the monolayer. As predicted for a negative control, *ACTA2* experienced downregulated expression across all timepoints up to seven days when DCs were cultured as microwell spheroids. *AXIN2* gene expression both increased steadily up to 5 days then drastically decreased at 7 days during HDPC spheroid culture in the microwell array platform. *WNT5A* exhibited similar gene expression patterns to *AXIN2* but much more modestly, as no significant changes between any of the timepoints were detected. *HIF1a* expression showed modest upregulation over 7 days, where there was more than a 2-fold increase compared to monolayer culture.

### 3.7. Dermal–Epidermal Composite Grafting

**Figure 11:**
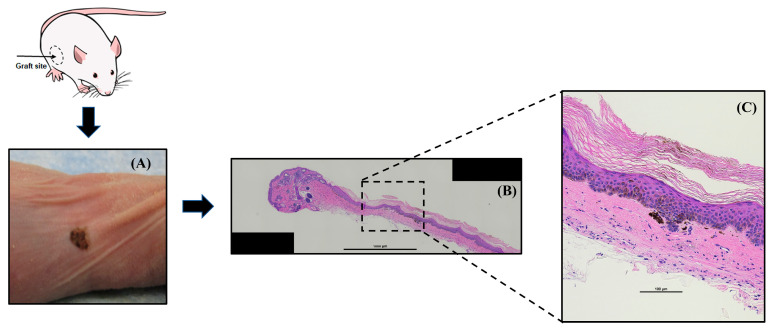
(**A**) Gross image of hyperpigmented human skin at immunocompromised mouse grafting site after proof-of-concept grafting microwell patterned HDPC spheroid DECs. (**B**) A 70× magnification image of H&E-stained skin graft from microwell spheroid DEC exhibited hair germ formation with stratified human epidermis and pigmented melanocytes in the basal epidermal layer; (**C**) in-set image at 390× magnification. Scale bar 1000 µm in (**B**) and 100 µm in (**C**).

As part of a proof-of-concept study, patterned microarray DECs were grafted onto a wound incision within the immunocompromised female mouse model and allowed to heal for 10 weeks after surgery. At 10 weeks, mice underwent gross skin assessment before being euthanized, followed by tissue harvesting for histological staining. Gross visual assessment indicated successful grafting with complete epithelialization and skin hypopigmentation present at the grafting site. Histological assessment using H&E staining exhibited the presence of a thickened human epidermis comprised of several layers of keratinocytes containing melanin pigment at the basal layers (Figure 11C). Due to a limited number of spatially patterned DEC samples prepared for xenografting, experimental endpoints such as graft size, epidermal thickness, and hair follicle neogenesis were not reported in this study.

## 4. Discussion

### 4.1. Engineering Biofabrication Strategies Improve Dermal–Epidermal Composite Design for Skin Grafting

To meet increased clinical demands, biofabrication strategies translated from laboratory to clinic are needed to rapidly and reproducibly create skin composites with patterned HDPC spheroids as models for improved wound repair and increased understanding of hair follicle neogenesis. Non-adherent microwell arrays offer advantages to pattern uniformly sized spheroids, with minimal cell aggregation or migration [30] compared to hanging drop methods [31]. In the microwell fabrication studies, the results validated PDMS as a suitable non-adherent cell substrate that enabled patterned HDPC spheroid formation from singularized cells, which did not occur in the 2% and 5% agarose hydrogel substrates. Patterned HDPC spheroids in the collagen matrix layer were then effectively transferred to the acellular layer of the dermal compartment before culturing in epidermalization media. At the air–liquid interface, HDPC viability within spheroids was maintained with some degree of cell migration through the collagen. While cell viability through spheroid depth was not reported, the literature reported spheroids with diameters greater than >500 µm having a necrotic core [32]. HDPC spheroids created in our work have spheroid diameters well below that, indicating that necrosis may not be an issue during DEC fabrication.

### 4.2. Effect of Spheroid Spatial Patterning on Genes Associated with Hair Follicle Neogenesis

#### 4.2.1. qPCR Gene Expression Across DC Cell Culture Platforms 

The effects of microwell patterned spheroids on an array of genes that function as a ligand or marker in HDPCs during hair follicle neogenesis were investigated. Growth of HDPC spheroids is known to partially restore hair inductivity lost during monolayer culture [22]. This is attributed to increased cell–cell contact within the HDPC spheroid to maintain its identity that would otherwise be lost as a monolayer [33]. Numerous signal transduction pathways in HDPCs are involved in the regulation of hair follicles [19,34]. In this study, *BMP6* was investigated due to its maintenance of HDPC function and niche identity towards hair follicle induction [35]. *BMP6* was significantly more upregulated in both hanging drop and microwell-templated spheroids compared to the monolayer (Figure 9). The Versican (*VCAN*) gene encodes the production of extracellular matrix proteins that help keep the dermal papilla intact and restore trichogenicity in vitro [33,36,37]. Versican is also a prominent HDPC marker during anagen, the most active phase of hair follicle regrowth that is lost during primary DP cell culture [33,38,39]. In our work, we aimed to recover hair induction using three-dimensional HDPC spheroids validated by *VCAN* upregulation. While *VCAN* expression in hanging drop spheroids decreased, *VCAN* gene expression significantly increased in microwell patterned spheroids compared to monolayer HDPC culture.

#### 4.2.2. qPCR Gene Expression Across DC Cell Culture Time Course

Next, we studied how HDPC spheroid culture time in non-adherent microwells influenced expression of critical HDPC-niche markers for hair follicle neogenesis. Our gene expression studies specifically focused on *BMP6* as part of the BMP signaling pathway. It was reported by Rendl et al. that *Bmp* genes in rodent models were critically important for hair follicle induction in DP cells [35]. Specifically, *Bmps* 2, 4, 6, and 7 induced significant fold changes in the expression of alkaline phosphatase specific genes in mouse DP cells [35]. For our study, *BMP6* gene expression was significantly greater 1 day after spheroid culture in the microwell system compared to the HDPC monolayer (Figure 10). *BMP6* expression fluctuated between 3 and 7 days before exhibiting lower expression than the 1-day timepoint but remained elevated compared to monolayer culture of HDPCs. *LEF1,* a HDPC gene that promotes hair differentiation [40], also fluctuated between 3 and 7 days but overall had significantly greater expression than the monolayer HDPCs. *ALPL* is a gene that encodes the production of alkaline phosphatase protein in HDPCs that increases beta-catenin/Wnt signaling for hair follicle neogenesis [41]. While trichogenicity in HDPCs diminishes with passage [35], our data demonstrated an overall increase of *ALPL* gene expression in 3D culture from 1 to 7 days (Figure 10).

An assortment of secondary genes involved in hair follicle signal transduction pathways [19,34] showed more modest changes in our gene expression data across time. The WNT signaling pathway is required for hair follicle development [19,42,43,44,45]. *Axin2* expression in mouse hair follicle stem cells [46] was reported to be induced by the *Wnt* pathway, where it functions as a negative regulator [47,48]. In HDPC spheroids, *WNT5A*, a gene that encodes for signature ligands in HDPCs [35], exhibited expression that was consistent over the timepoints as *AXIN2* gene expression increased, particularly at 1 and 3 days (Figure 10). This finding suggests that the presence of *WNT5A* may impact increased “stemness” of HDPCs in the hair follicle bulge region over time during in vitro dermal component fabrication. *HIF1α*, part of the pI3K/AKT pathway, is induced by hypoxia in three-dimensional spheroids and promotes trichogenicity [49]. Increased *HIF1α* expression reported in HDPC spheroids after 3 days in culture suggested the spheroid interior is hypoxic [29]. Our data show a similar trend, with microwell HDPC spheroids significantly upregulated *HIF1a* expression after 7 days, suggesting increased hypoxia and trichogenicity.

By day 5, switching from HDP to epithelial Prime Airlift media significantly altered the expressions of six of the nine tested genes (*FGF7*, *VCAN*, *AXIN2*, *HIF1A*, *BMP6*, and *ALPL*) in the dermal composite compared to day 3. However, changes in the affected HDPC niche markers are noted but do not always directly translate into protein expression to improve trichogenicity or have post-translational modification alterations that inhibit hair follicle activity. This is likely due to a complicated set of factors during cell culture that will need to be further investigated in future work. It is possible that the lack of feedback from keratinocytes may influence dermal papilla gene expression monitored in our studies, as keratinocytes were added to the constructs used for grafting. Since keratinocytes are known to work in concert with DP cells during regenerative hair follicle cycling [18,50,51], it is likely that the gene expression results reported here would change due to the influence of keratinocytes on DP cell physiology. A recent study highlighted that adult keratinocyte-conditioned media improves DP cell hair inductivity and proliferation [52]. In addition, as the cells spread out of the spheroids (Figure 7), their gene expression may begin to revert to that seen in the monolayer, which may explain the decrease in *BMP6*, *VCAN*, and *FGF7* expression in the later timepoints relative to the initial expression detected in the microwell spheroids (Figure 10). Additional future in vitro studies include performing immunohistochemistry staining of spheroids in microwell DEC grafts to examine changes in protein and hair follicle marker expression. Taken together, the gene expression results reveal that HDPC spheroids grown in 3D culture using non-adherent microwell arrays broadly impart a positive effect on hair follicle induction genes within the dermal papillae.

### 4.3. Effect of Spheroid Spatial Patterning on Dermal–Epidermal Composite Grafting

DECs with spatially patterned HDPC spheroids using non-adherent microwell arrays were successfully grafted into a xenograft mouse model as a means to evaluate the ability of these in vitro manufactured DECs to serve as skin grafts. As a proof-of-principle study to examine graft performance, histology at 10 weeks exhibited gross presence of pigmented skin and a thickened human epidermis at the graft site (Figure 11). Though the two grafts did not exhibit observable hair regrowth, extending the study beyond 10 weeks to integrate HDPCs and HKs into mouse skin may improve the likelihood of initiating hair follicle neogenesis. The results of the grafting study while encouraging are in the preliminary stages. To better evaluate the graft performance of spatially patterned DECs using non-adherent microarrays, more robust rodent model studies are needed to address the current sample size limitations. In addition, experiments investigating histological and immunohistochemistry endpoints of hair follicle and wound repair markers are needed to validate graft performance. Bioactive peptides screened from our previous work have revealed increased endothelial proliferation in vitro, improving microvasculature of the dermal component in vivo to enhance wound re-epithelialization and hair folliculogenesis in xenografted mouse models [15,16]. Future work integrating these peptides into microwell patterned spheroids could further improve dermal papilla trichogenicity, epidermal thickness, and hair follicle neogenesis in grafted DECs.

## 5. Conclusions

We used 3D printing as a feasible biofabrication strategy to develop non-adherent microwell arrays to rapidly and reproducibly pattern HDPC spheroids from singularized cells in dermal–epidermal composites. Hair follicle stem cell signaling factors and HDPC niche markers are more amenable in a 3D microwell microenvironment versus 2D monolayer culture. As a proof of concept, DECs were successfully grafted and initiated re-epithelialization of a new human epidermis in a mouse model for up to 10 weeks post-grafting. Non-adherent PDMS microwell arrays offer a tunable platform to vary spheroid number, arrangement, and size in a reproducible manner. This platform has potential to create experimental 3D HDPC spheroid models architecturally similar to the base of human hair follicles. Novel biofabrication approaches that leverage patterned three-dimensional dermal papilla cells in skin composites can advance dermal–epidermal composite design to improve wound healing and hair follicle neogenesis, meeting the accelerating demands of wound care.

## Data Availability

Data supporting the conclusions are available upon request from the authors.
